# Filamentous Fungi Infections: Yet Another Victim of COVID-19?

**DOI:** 10.3390/life13020546

**Published:** 2023-02-15

**Authors:** Anca Cighir, Anca Delia Mare, Teodora Cighir, Răzvan Lucian Coșeriu, Camelia Vintilă, Adrian Man

**Affiliations:** 1 Department of Microbiology, George Emil Palade University of Medicine, Pharmacy, Sciences and Technology of Târgu Mures, 38 Gheorghe Marinescu Street, 540139 Târgu Mures, Romania; 2 Doctoral School of Medicine and Pharmacy, George Emil Palade University of Medicine, Pharmacy, Sciences and Technology of Târgu Mures, 38 Gheorghe Marinescu Street, 540139 Târgu Mures, Romania; 3 Mureș Clinical County Hospital, 1 Gheorghe Marinescu Street, 540103 Târgu Mures, Romania

**Keywords:** filamentous fungi, *Aspergillus* spp., COVID-19

## Abstract

Filamentous fungi have always been a matter of concern in the medical field, but nowadays, due to an increase in the risk factors and the added infections with the SARS-CoV-2 virus, they are slowly but surely emerging as a dangerous health threat worldwide. Our study aims to estimate the incidence of mold infections in central Romania, as well as assess the impact the pandemic had on them while evaluating other parameters such as age, associated bacterial and fungal infections and comorbidities. Purulent discharge and respiratory secretion specimens were collected and analyzed over a period of 10 years. A total of 68 samples tested positive for molds, with an increased number of positive samples during the pandemic. The highest number of specimens came from the outpatient department, followed by medical wards, with the most common filamentous fungus being *Aspergillus section Flavi*. Associated diseases included affections of the respiratory system, followed by the cardiovascular system and people who suffered from a viral infection with SARS-CoV-2, and they were mostly present in seniors. The most common associated infections were with *Staphylococcus aureus* and *Candida* nonalbicans. A statistically significant correlation was found between the association of mold infection and SARS-CoV-2 and an increase in mortality.

## 1. Introduction

Fungal infections are one of the most difficult-to-manage diseases known to humankind [1]. It is estimated that there are around 6 million fungal species on our planet, but only several hundred of them are able to cause disease in humans, and even fewer can infect healthy people [1,2]. A limited number of fungi are primary pathogens, being able to cause infection both in immunocompetent and immunosuppressed patients. In contrast, the majority of filamentous fungi that cause human infections are opportunistic pathogens and require certain conditions to be met before they can cause infection (e.g., immunosuppressed host) [3].

In the past, fungal infections were mainly caused by species from the genus *Candida* spp., but lately, due to an increase in life expectancy and, along with it, the associated risk factors, more and more pathogens have emerged. Therefore, other fungi such as dimorphic fungi (e.g., *Coccidioides* spp., *Blastomyces* spp., *Histoplasma* spp. and *Paracoccidioides* spp.) and hyaline molds (e.g., *Aspergillus* spp., *Zygomycetes* and *Fusarium* spp.) are slowly taking over [4].

Each fungal species causing infection has its own specific risk factors and, therefore, epidemiology associated with it. In certain parts of the world, fungal infections are linked to the presence of an underlying disease (e.g., a higher prevalence of cryptococcal infections and pneumocystis infections in areas with a high prevalence of HIV infection) [5]. Dimorphic fungi are endemic to tropical areas and are frequently related to professional exposure (e.g., forest rangers, miners, cavers, and people who work in construction or farming) and outdoor recreational activities (e.g., camping and fishing) [5,6]. Mold infections are usually associated with the presence of an underlying immunosuppressive factor (e.g., uncontrolled diabetes, hematological malignancy, solid organ transplantation, hematopoietic cell transplant, solid tumors, HIV/AIDS or inherited immunodeficiency) [5]. 

Since it was first recognized as a threat back in 2019, the severe acute respiratory syndrome coronavirus 2 (SARS-CoV-2) and the disease caused by it, COVID-19, became one of the main research topics worldwide: starting from the understanding and management of this virus and the disease it causes, to the prevention of its spread, associated infections, treatment and vaccination [7]. Most of those factors remain unclear even nowadays. As more and more diseases were easily acquired due to the fact that the organism was weakened by the viral infection or became influenced in their evolution by this virus, research regarding this topic became imperiously needed. 

Very few studies are currently available regarding filamentous fungi prevalence in Romania [8,9]. Furthermore, since the last published studies, the COVID-19 pandemic began, giving the human population an additional risk factor that could increase the chance of acquiring these mycoses.

The purpose of our study is to evaluate the prevalence of filamentous fungi infections in a tertiary care hospital in Târgu Mureș, Romania, as well as evaluate the correlation between age, risk factors (underlying diseases) and their association with the presence of those fungal pathogens. Furthermore, the influence of the COVID-19 pandemic on the number of mold infections was studied.

## 2. Methods

A single-center retrospective observational study in which data was collected from the Clinical County hospital of Târgu Mureș was conducted between 1 January 2012 and 1 November 2022. The samples consisted of purulent secretions (e.g., ascitic fluid, conjunctival discharge, ear discharge, nail samples) and respiratory secretions (e.g., sputum, tracheal aspirate, bronchial lavage). For patients with multiple samples sent for evaluation in the same time frame, only the first one was taken into consideration. Incomplete entries were also eliminated from the data analysis. 

For each sample, several parameters were assessed: age, gender, department, sample type, associated bacterial or fungal infections and risk factors for developing those infections (e.g., associated diseases, immunosuppression, COVID-19 infection). 

The processed samples belonged to different wards—medical wards (e.g., internal medicine, nephrology, cardiology, oncology, infectious diseases, ophthalmology, pneumology), surgical wards (e.g., general surgery, plastic surgery), Intensive care units—ICU (divided as COVID-19 ward and non-COVID-19 ward once the pandemic started) and the outpatient department.

For respiratory secretions, a quality triage of the samples was also performed. Tracheal aspirates and bronchial lavages were considered suitable for processing by default due to the aseptic way of harvesting the sample. In the case of sputum, the evaluation was performed based on microscopical examination directly from the pathological product, and it consisted of using the Bartlett score (Q), which assessed the presence of leukocytes, squamous epithelial cells and mucus under the microscope in 10 low power fields (LPF). The three criteria were evaluated and pointed out accordingly:

- <10 neutrophils/LPF = 0, 10–20 neutrophils/LPF = +1, >20 neutrophils/LPF = +2;
- <10 squamous epithelial cells/LPF = 0, 10–25 squamous epithelial cells/LPF = −1, >25 squamous epithelial cells/LPF = −2;
- presence of mucus = +1.

A final score of less than one implied significant contamination of the sputum sample with squamous cells of the superior respiratory tract, therefore making the samples unsuitable for processing. A final score of one or above meant a high number of inflammatory cells, making the sample suitable for further investigations.

The samples were transported to the laboratory and cultured on the appropriate culture media: blood agar, Mannitol Salt agar, lactose agar, Chocolate blood agar and Sabouraud dextrose agar, then incubated at 32 °C for 24–72 (or up to 1–2 weeks, if deemed necessary). If colonies specific for molds were detected on any of the culture media, they were further isolated on Sabouraud dextrose agar, and the fungal genus was identified based on the macroscopical aspect as well as microscopical examinations using lactophenol cotton blue staining.

In order to differentiate between colonization and infection, in the case of respiratory samples, several criteria were taken into consideration: if the symptoms of the patients were characteristic of a mycotic infection (dyspnea, fever), if any specific modifications were present on the paraclinical examinations (nonspecific infiltrates, nodular or cavitary lesions) and the presence of risk factors that would lead to the suspicion of such mycosis. To further aid our diagnosis, the sample type was taken into consideration: if the filamentous fungi were isolated from a deep respiratory sample like tracheal aspirate, where the risk of contamination with normal flora of the upper respiratory tract is minimal, the microorganism was considered an etiological agent of the infection; if the fungus was isolated from a sputum sample, the before mentioned criteria were taken into consideration, and another sample was requested in order to confirm the infection.

Clinical and paraclinical data was recorded in the spreadsheet software, anonymized and statistically analyzed. The Shapiro–Wilk normality test was applied, and non-parametric tests were performed where necessary. To detect significant differences between data, Fisher's exact test (when at least one of the frequency values in the table was less than five) or Chi test was applied for all contingency tables. The alfa value was set to 0.05. 

## 3. Results

During the ten-year period, a total of 95 positive samples of 23,777 processed samples (0.40%) were detected. After the removal of duplicated and incomplete patient data entries, a number of 68 positive samples (0.29%) were further analyzed. 

Among them, 41 positive purulent discharges (0.17% from the total number of processed samples, 0.20% of the purulent discharge samples) and 27 positive respiratory secretions (0.11% from the total number of processed samples, 0.79% of the respiratory secretions) were identified. 

The lowest and highest number of positive samples for purulent discharge was noticed in 2012 (0.05%; *n* = 1) and 2021 (0.46%; *n* = 9), respectively, while for respiratory secretions in 2013, 2019, 2020 (0%; *n* = 0) and in 2017 (4.85%; *n* = 5). 

**Figure 1:**
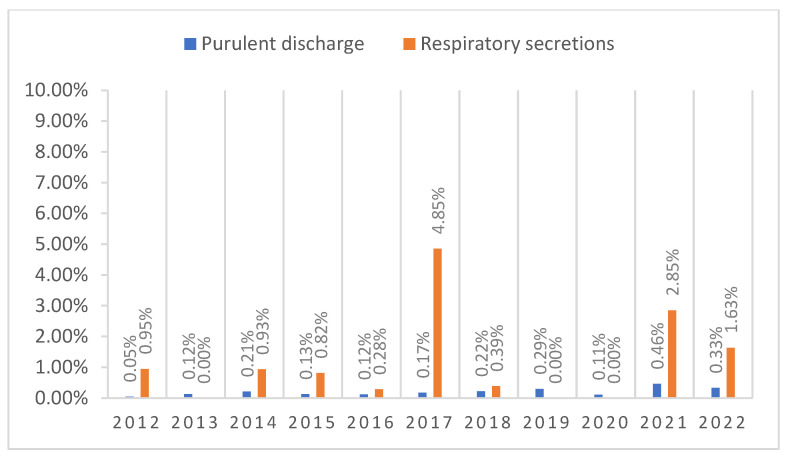
Positive sample distribution by years.

A spike of positive cases in the respiratory secretions group was noted during 2021, when the COVID-19 pandemic was still ongoing, as 7 out of the 9 positive samples (77.8%) originated from the COVID-19 ICU (Figure 1). 

Table 1 summarizes the number of samples taken during each of the years that were included in the study as well as the number of positive samples, dividing it by pre-pandemic and post-pandemic time criteria. As can be seen, the number of samples taken varied greatly, especially during the pandemic time frame. 

In the pre-pandemic years, even though the number of samples processed each year was high, the number of positive samples was still low, and it mostly consisted of specimens from the outpatient department, ICU and Oncology department, from patients who had associated risk factors and an advanced age. The sample types that were positive for fungal infection also differed, as a higher number of purulent discharge samples was positive (*n* = 18) than respiratory secretions (*n* = 14). A spike in the number of samples can be noticed in 2017, directly related to the increased number of samples processed that year. 

During the pandemic years, the percentage of positive samples increased. This was caused not only by the decrease in the number of samples taken but an increase in the number of positive samples as well. In the first two years of the pandemic, due to the number of departments being closed and the restructuring of the hospital wards, the number of patients admitted to the hospital as well as the number of processed samples, dropped. However, even taking this into consideration, the number of samples that tested positive for molds was still considerably higher than before the pandemic. Furthermore, even though the number of purulent discharge samples (*n* = 23) was still higher than that of respiratory samples (*n* = 13), the latter all came from COVID-19-positive patients (with one exception) and were taken in 2021, when the pandemic was still at its peak. 

A comparison regarding statistical significance in the increase or decrease in the number of positive samples can be seen in Table 2. A statistical significance can be noted mostly between the number of samples taken in the pre-COVID-19 pandemic years and during the pandemic years. 

From the purulent discharge samples, the one that tested positive for mold infections the most was the ear discharge, with a 32.35% positive rate (*n* = 22), while from the respiratory secretions, the orotracheal tubes were positive in 20.59% of cases (*n* = 14). The most common filamentous fungus in both purulent discharge and respiratory secretions was *Aspergillus section Flavi*, with 22.06% positive results for purulent discharge (*n* = 15) and 92.59% positive rate of the respiratory samples (*n* = 25).

From the positive samples, different molds were identified. The most common one by far was *Aspergillus* spp. (79.41%; *n* = 54)—out of which *Aspergillus section Flavi* (58.82%; *n* = 40) and *Aspergillus section Nigri* (20.59%; *n* = 14), followed by other genra such as *Fusarium* spp. (11.76%; *n* = 8), *Trichophyton* spp. (4.41%; *n* = 3) and *Alternaria* spp.,* Acremonium* spp. and *Epidermophyton* spp. (1.47%; *n* = 1)—Table 3. In COVID-19 patients, the most commonly isolated fungus from respiratory secretions was *Aspergillus section Flavi* (78.57%; *n* = 11).

Regarding sample distribution based on wards, 32.35% of the total positive samples originated from the outpatient department (*n* = 22), 27.94% from medical wards (highest: cardiology—7.35%, *n* = 5; lowest: infectious diseases, internal medicine, nephrology, ophthalmology, pediatrics, tuberculosis ward—1.47%, *n* = 1), 25% from intensive care units (highest: COVID-19 ward with 14.71% positive samples, *n* = 10) and 14.71% originated from surgical wards (7.35% positive samples on both wards, *n* = 5) (Table 4).

Even though it had no statistical significance (*p* = 0.946), age distribution still showed an increase in the number of cases proportionally with the age increase: while children (under 14 years old) and young adults (15–29 years old) had an incidence of 2.94% (*n* = 2) and 4.41% (*n* = 3) respectively, adults (30–64 years old) and seniors (>65 years old) had a much higher incidence, 35.29% (*n* = 24) and 57.35% (*n* = 39) respectively. Gender distribution showed no significant statistical difference (*p* = 0.2), with 44.12% (*n* = 30) of the positive cases being females and 55.88% (*n* = 38) of cases being males.

Age distribution was also compared based on the samples that were positive. Even though there was no significant statistical difference noted between the two (*p* = 0.236), the mean age (66.96 +/− 17.90 with a minimum of 12 and maximum of 71) of the patients who had molds in respiratory tract secretions was higher than in those who had purulent discharge as a sample (61.85 +/− 16.82, with a minimum of 12 and maximum of 92).

Out of the total number of positive cases, 64.71% (*n* = 44) of the patients had an associated bacterial or fungal infection. From them, 39.71% (*n* = 27) people had an associated bacterial infection, 39.71% (*n* = 27) people had a fungal infection, and 16.18% (*n* = 11) people had both bacterial and yeast infections. An overview of the most commonly associated pathogens based on age group can be seen in Table 5. The most frequently associated bacterial infection was *Staphylococcus aureus* (14.71%; *n* = 10), followed by *Pseudomonas* spp. (7.35%; *n* = 5), *Klebsiella* spp. (5.88%; *n* = 4) and *Streptococcus* spp. (5.88%; *n* = 4) while the most common fungal infection was with *Candida* non-albicans (30.88%; *n* = 21), followed by *Candida albicans* (14.71%; *n* = 10). 

*Staphylococcus* spp. was found in both purulent discharge and respiratory secretions equally (*n* = 5), *Pseudomonas* spp. was more common in puss samples (*n* = 4) than in respiratory samples (*n* = 1) while *Klebsiella* spp. was dominant in respiratory secretions (*n* = 3). Associated fungal infections with *Candida albicans* were mostly found in respiratory secretions (*n* = 9), while *Candida* non-albicans were found in similar degrees in both purulent discharge (*n* = 10) and respiratory secretions (*n* = 11).

In the case of SARS-CoV-2 positive patients, associated fungal infections were more common than bacterial infections, with an equal number of infections with *Candida albicans* and nonalbicans species (28.57%; *n* = 4). Regarding bacterial co-infections, the most common one was *Pseudomonas aeruginosa* (14.29%; *n* = 2)—Table 6.

Because mold infections are known to be associated with the presence of an underlying immunosuppressive factor, our study also took comorbidities into consideration. The number of associated diseases varied, with 69.12% of the patients having comorbidities (*n* = 47). The mean value was 1.30 +/− 1.319 comorbidities/person. The minimum number of associated diseases in a patient was zero, while the highest number was five. A summary of the most common risk factors that were evaluated can be found in Table 7.

The highest number of associated comorbidities was present in seniors, followed by adults, youth and children. The most targeted organ system was the respiratory system, followed by the cardiovascular system and people who suffered from a viral infection with SARS-CoV-2. Other affected organs, but in a lower number, included the digestive system, the skin, kidneys and malignities. 

Associated diseases consisted of pathologies of the:

- respiratory tract: bronchopneumonia, chronic obstructive pulmonary disease, pulmonary abscesses, tuberculosis, acute respiratory insufficiency, mucoviscidosis;
- cardiovascular system: heart failure, atherosclerosis, hypertension, atrial fibrillation;
- digestive system: Crohn’s disease, hepatic hydatic cyst, acute pancreatitis, viral hepatitis;
- skin: psoriasis, skin and subcutaneous tissue infections, gangrene;
- malignities: adenocarcinoma of the colon, bronchi and laryngeal tumors;
- kidneys: calciphylaxis, renal failure.

A notable subcategory of patients is those who tested positive for SARS-CoV-2 infection. As can be seen from Table 8, in their case, the most commonly associated diseases consisted of affections of the cardiovascular system (64.29%; *n* = 9), followed by the respiratory system (50%; *n* = 7), diabetes and kidney disease (21.43%; *n* = 3).

The mortality among patients who developed invasive mold infections was 19.12% (*n* = 13), out of which 10 cases were in patients who previously tested positive for SARS-CoV-2 infection (71.43%). In the positive sample group, a statistically significant correlation was found between the association of mold infection and SARS-CoV-2 and an increase in mortality (*p* < 0.0001; OR = 24.5).

## 4. Discussion

This retrospective observational study describes the epidemiology of filamentous fungal infections in a tertiary care hospital over a period of ten years. To the best of our knowledge, this research is one of the first studies related to the incidence of invasive mold mycosis in central Romania. 

In recent years, an increase in the number of fungal infections with both yeasts and molds can be observed. One of the main causes of this is the continuously increasing population of immunocompromised patients who undergo different treatments such as hematopoietic stem cell transplantation (HSCT), solid organ transplantation (SOT) or treatments with immunomodulatory agents [10]. 

The number of studies talking about the incidence of mold infections in Romania is limited. A recent article that shows an overview of those infections was published in 2018 by Mareș et al. [8], which analyzed not only the number of yeast infections but molds as well. The authors measured the fungal burden of several infections in Romania in 2016. For invasive aspergillosis, the mentioned rate was 7.7/100,000 inhabitants. Based on that rate and the total population of Târgu Mureș (approximately 150,000 inhabitants in 2016), the estimated rate for our city would be 11.55 cases of invasive aspergillosis/150,000 inhabitants. In contrast, our study showed a much smaller incidence of only 2 cases/150,000 inhabitants (2016). If a 10-year mean of all infections with *Aspergillus* spp. was calculated, the incidence would be 5.4 cases/150,000 inhabitants, closer to the estimated rate but still low in comparison. 

Bongomin et al. published a study on the global incidence of different mycoses and their impact on human health [11]. The authors estimated the incidence of invasive aspergillosis based on data from 40 countries and calculated an average incidence of 4.10 cases/100,000 inhabitants (6.15/150,000 inhabitants), a value that is close to the mean value of 5.4 cases/150,000 inhabitants that we obtained. Regarding other molds causing infections in immunocompromised patients, *Fusarium* spp. had an infection rate of 1.7% of positive patients with filamentous mycoses [4]. In our study, the mean incidence was much higher, showing a surprising 11.76% (*n* = 8) of total positive samples in the time span of 10 years. Furthermore, in our study, an even more surprising aspect regarding the incidence of *Fusarium* spp. is the difference in the number of cases before the pandemic (when only two cases were found) and during the pandemic, when six cases of fusariosis were diagnosed. All isolates originated from purulent discharge, with one exception, when the fungus was found in a corneal scraping sample. Even though none of the patients suffered from the viral infection with SARS-CoV-2, six out of eight patients presented other associated risk factors. 

When discussing why the number of cases suddenly spiked in recent years, two factors could be taken into consideration. First of all, in Romania, part of the population still lives in the countryside and has agriculture as their main source of food and income. As *Fusarium* spp. can be found in soil, water, and air and is recognized as a plant pathogen, it can easily contaminate open wounds and cause infection when suitable conditions appear [12]. Secondly, the influence of the pandemic should be taken into consideration. Kalanj et al. published an article about the impact the COVID-19 pandemic had on the medical system [13]. The publication shows a decrease in the number of patients who addressed the hospitals for medical services during the pandemic of up to 51%, and they assume there are several reasons why this happened: the lockdown, the quarantine, the reorganization of hospital wards, the redistribution of the medical staff towards departments where they were imperiously necessary and the reluctance of people to seek hospital care due to the fear of acquiring the disease. Thus, we concluded that also, in our case, most people who presented different medical problems were not able to get any specialized medical help until the evolution of their disease complicated. Thus, they were forced to present directly to the emergency room. Moreover, the impossibility of traveling long distances during the lockdown in order to reach a medical unit contributed to the poor outcome of the disease. 

Besides the widely studied risk factors for developing invasive mold infections, starting in 2020, a new health threat appeared, the SARS-CoV-2 virus. During the pandemic, one of the biggest concerns of the medical staff, besides the appropriate treatment for the disease, was the associated infections complicating the evolution of the patients. Commonly found pathogens were bacteria (e.g., *Streptococcus pneumoniae* and *Mycoplasma pneumoniae*), as well as viruses and with a lower but significant incidence, yeasts (e.g., *Candida* spp.) and molds (e.g., *Aspergillus fumigatus* followed closely by *Aspergillus flavus*) [14].

Multiple articles regarding the association between invasive mold infections (especially aspergillosis) and severe or critical forms of COVID-19 were published [14,15,16,17,18,19,20,21,22]. Similarly to our cases, when COVID-19 was associated with aspergillosis, the mortality rate was very high [18]. Something else worth noting is that, in the case of association with SARS-CoV-2, the conventional risk factors are not needed anymore for the development of invasive aspergillosis [7]. In our case, all patients that presented the viral infection also had between 1–5 comorbidities, more frequently of the cardiovascular or respiratory system.

Patients with SARS-CoV-2 infection can develop invasive forms of aspergillosis mostly due to the damage of the epithelium and immune dysregulation leading to tissue invasion associated with the immunomodulatory therapy those patients receive [23]. One of the challenges regarding the diagnosis of invasive aspergillosis in the context of viral infections still remains to this day the difficulty in differentiating between colonization and infection and whether one should always treat *Aspergillus* spp. once found in the respiratory tract samples [15,18]. 

According to Machado et al. [18], a few significant criteria should be taken into consideration when trying to establish a diagnosis: the presence of symptoms typical of respiratory mycosis (e.g., fever, pleuritic pain, dyspnea), modifications of computed-tomography or X-ray and the presence of several risk factors apart from the SARS-CoV-2 infection (e.g., neutropenia or hematological or oncological diseases). Colonization is considered when none of the before mentioned criteria is present, and the mold is isolated from the sample. Another factor that contributed to the separation between colonization and infection was the evolution of the patient under treatment. 

Cadena et al. [23] also mentioned in their study that the factor that should be taken into consideration when differentiating between colonization and infection is the sample type: while sputum samples are easier to obtain, they are also more prone to putting into light a colonization; bronchoalveolar lavage is harder to sample, but it can be more suggestive of an aspergillar infection. Molecular diagnosis (polymerase chain reaction) can also be a good aid in detecting filamentous fungi, but it can differentiate between colonization and infection only when specific clinical settings are present.

In our case, unfortunately, little data is available regarding the symptoms of the patients, as the acknowledged symptoms are mostly related to their viral infection, but taking their evolution into consideration, the two people who had a favorable evolution towards healing could be considered cases of colonization and not infection.

The point of differentiating between colonization and infection when isolating a microorganism from a respiratory specimen should also be taken into consideration when discussing associated infections, especially co-infections with *Candida* spp. As in the case of filamentous fungi, respiratory infections with yeasts can only be confirmed in selected cases (e.g., when symptoms and radiological changes are present, when the patient has risk factors) or by taking an appropriate sample [24]. We have to note that *Candida* pneumonia is very rare, this yeast being involved mostly in invasive infections in risk patients, which is not our case [25]. Nevertheless, the elements that could help the diagnosis of yeast infections are similar to the ones of filamentous mycosis, especially if they are not invasive [26,27]. Thus, the identification of the real fungal etiological agent is, unfortunately, hard to be established, and the patients are eventually treated for both in most cases.

Generally known risk factors for developing mycoses were also evaluated in our study. Lass-Flörl et al. and Egbuta et al. mentioned in their articles [4,28] some of the most commonly associated diseases: hematological malignancy, affections of the respiratory system (e.g., chronic obstructive pulmonary disease, asthma), solid tumors, hematopoietic stem cell transplant recipients, neutropenic patients, immunocompromised patients, skin lesions, diabetes mellitus. Cadena et al. [23] suggested that more factors, such as patients within the intensive care unit, immunosuppressive treatments (e.g., immunomodulators, corticotherapy) and patients who suffer from respiratory viral infections (such as SARS-CoV-2, Influenza virus infection) can be considered a potential population at risk. Similar factors were also present in our study group and were thus associated with the presence of the fungal disease. 

In contrast, a limitation of our research consists of the fact that our hospital does not collect patient samples from the hematology department, hematological diseases being one of the main risk factors for developing those types of infections. Therefore, if those samples could be included, the incidence of filamentous mycoses could be slightly higher. However, even taking this into consideration, our estimated incidence was still similar to the data reported in the literature.

Very limited information is available regarding the association of different bacteria and fungi to filamentous fungi infection. For example, Rawson et al. recently described the bacterial and fungal infections that are associated with SARS-CoV-2 infection; one case showed an association of *Aspergillus flavus*, *Klebsiella pneumoniae* and multidrug-resistant *Acinetobacter baumannii* [29]. In our study, patients presented co-infections with yeasts rather than with bacteria. 

## 5. Conclusions

Filamentous mycoses are slowly but surely becoming a feared health threat worldwide. As life expectancy increases, new risk factors and new treatments appear, and the population is slowly shifting towards a more susceptible target for those types of infections.

Since the beginning of the COVID-19 pandemic, a novel risk factor has appeared, therefore increasing the number of infections with those pathogens even more. Thus, besides learning how to manage the viral disease, medical staff around the world had to learn when to look for associated infections with pathogens that were rarely found beforehand, as well as diagnose and treat them. If, in the past, multiple risk factors were required to acquire a mold infection, now just acquiring the viral infection could weaken the organism enough to cause such a mycosis. 

As it can be seen from our study, in recent years, the number of filamentous fungi raised slowly but surely. Thus, more studies related to the epidemiology, diagnosis and treatment of those infections are needed in order to cover this knowledge gap and further aid the medical personnel in treating those patients. 

Unfortunately, even nowadays, when more and more antifungal treatments become available, filamentous mycoses are still associated with increased morbidity and mortality due to their association with increased age and an already diseased organism. Even in situations like these, fast and accurate diagnosis, as well as aggressive treatments, are still essential tools for good clinical outcomes.

## Figures and Tables

**Table 1:** Positive sample distribution by years.

	Before COVID-19 Pandemic (*n* = 32)							During COVID-19 Pandemic (*n* = 36)				Total
	2012	2013	2014	2015	2016	2017	2018	2019	2020	2021	2022	
Positive samples/year (n)	3	2	5	4	3	9	6	6	2	18	10	68
Total processed samples/year (n)	2415	1913	1661	1802	2048	2460	1325	1433	1511	1640	1587	23,777
**%**	0.12	0.10	0.30	0.22	0.15	0.37	0.45	0.42	0.13	1.10	0.63	0.29

**Table 2:** Statistical significance of the increase or decrease in the number of positive samples/year. The statistically significant *p* values are marked in bold.

Before COVID-19 Pandemic									During COVID-19 Pandemic		
	2012 (*n* = 3)	2013 (*n* = 2)	2014 (*n* = 5)	2015 (*n* = 4)	2016 (*n* = 3)	2017 (*n* = 9)	2018 (*n* = 6)	2019 (*n* = 6)	2020 (*n* = 2)	2021 (*n* = 18)	2022 (*n* = 10)
2012	-	*p* = 0.849 OR = 1.188	*p* = 0.371 OR = 0.411	*p* = 0.697 OR = 0.151	*p* = 0.839 OR = 0.847	*p* = 0.157 OR = 0.338	*p* = 0.106 OR = 0.273	*p* = 0.138 OR = 0.295	*p* = 0.944 OR = 0.938	***p* < 0.0001** **OR = 0.112**	***p* = 0.013** **OR = 0.196**
2013	-	-	*p* = 0.344 OR = 0.346	*p* = 0.629 OR = 0.470	*p* = 0.710 OR = 0.713	*p* = 0.159 OR = 0.285	*p* = 0.109 OR = 0.230	*p* = 0.137 OR = 0.248	***p* = 0.049** **OR = 0.197**	***p* = 0.0002** **OR = 0.0943**	***p* = 0.018** **OR = 0.165**
2014	-	-	-	*p* = 0.902 OR = 1.357	*p* = 0.513 OR = 2.058	*p* = 0.937 OR = 0.822	*p* = 0.706 OR = 0.663	*p* = 0.806 OR = 0.718	*p* = 0.527 OR = 2.278	***p* = 0.011** **OR = 0.272**	*p* = 0.261 OR = 0.476
2015	-	-	-	-	*p* = 0.865 OR = 1.516	*p* = 0.575 OR = 0.605	*p* = 0.418 OR = 0.489	*p* = 0.495 OR = 0.529	*p* = 0.846 OR = 1.679	***p* = 0.002** **OR = 0.200**	*p* = 0.114 OR = 0.350
2016	-	-	-	-	-	*p* = 0.257 OR = 0.399	*p* = 0.179 OR = 0.322	*p* = 0.223 OR = 0.348	*p* = 0.911 OR = 0.0123	***p* = 0.0003** **OR = 0.132**	***p* = 0.032** **OR = 0.231**
2017	-	-	-	-	-	-	*p* = 0.892 OR = 0.807	*p* = 0.797 OR = 0.873	*p* = 0.294 OR = 2.771	***p* = 0.008** **OR = 0.330**	*p* = 0.334 OR = 0.579
2018	-	-	-	-	-	-	-	*p* = 0.891 OR = 1.082	*p* = 0.211 OR = 3.432	*p* = 0.081 OR = 0.409	*p* = 0.694 OR = 0.717
2019	-	-	-	-	-	-	-	-	*p* = 0.255 OR = 3.172	*p*= 0.053 OR = 0.378	*p* = 0.583 OR = 0.663
2020	-	-	-	-	-	-	-	-	-	***p* = 0.001** **OR = 0.119**	*p* = 0.052 OR = 0.209
2021	-	-	-	-	-	-	-	-	-	-	*p* = 0.214 OR = 1.750

**Table 3:** Distribution of different molds in the positive samples.

Fungal Genus	Purulent Discharge		Respiratory Secretions	
	*n*=	Percentage	*n*=	Percentage
*Acremonium* spp.	1	1.47%	0	0.00%
*Alternaria* spp.	0	0.00%	1	3.70%
*Aspergillus* spp.	28	41.18%	26	96.30%
*Aspergillus* section *Flavi*	15	22.06%	25	92.59%
*Aspergillus* section *Nigri*	13	19.12%	1	3.70%
*Epidermophyton* spp.	1	1.47%	0	0.00%
*Fusarium* spp.	8	11.76%	0	0.00%
*Trichophyton* spp.	3	4.41%	0	0.00%
Total	41	60.29%	27	39.71%

**Table 4:** Distribution of samples based on wards.

	Department	Purulent Discharge		Respiratory Secretions	
		*n*=	Percentage	*n*=	Percentage
Medical wards	Cardiology	3	4.41%	2	2.94%
	Infectious diseases	3	4.41%	0	0.00%
	Internal medicine	0	0.00%	1	1.47%
	Nephrology	0	0.00%	1	1.47%
	Ophthalmology	1	1.47%	0	0.00%
	Oncology	1	1.47%	3	4.41%
	Pediatrics	0	0.00%	1	1.47%
	Pneumology	1	1.47%	1	1.47%
	Tuberculosis ward	0	0.00%	1	1.47%
Surgical wards	Plastic surgery	5	7.35%	0	0.00%
	Surgery	4	5.88%	1	1.47%
OPD	Outpatient department	22	32.35%	0	0.00%
ICU	non-COVID-19 ICU	1	1.47%	6	8.82%
	COVID-19 ICU	0	0.00%	10	14.71%
	Total	41	60.29%	27	39.71%

**Table 5:** Mold-associated infections.

	Microorganism	Child (*n* = 2)		Youth (*n* = 3)		Adult (*n* = 24)		Senior (*n* = 39)	
		*n*=	%	*n*=	%	*n*=	%	*n*=	%
*Bacterial infections*	*Acinetobacter* spp.	0	0.00%	0	0.00%	1	2.27%	2	4.55%
	*Corynebacterium* spp.	0	0.00%	0	0.00%	0	0.00%	1	2.27%
	*Citrobacter* spp.	0	0.00%	0	0.00%	0	0.00%	1	2.27%
	*Enterobacter* spp.	0	0.00%	0	0.00%	0	0.00%	2	4.55%
	*Escherichia coli*	0	0.00%	0	0.00%	2	4.55%	0	0.00%
	*Enterococcus* spp.	0	0.00%	0	0.00%	1	2.27%	1	2.27%
	*Proteus* spp.	0	0.00%	0	0.00%	0	0.00%	1	2.27%
	*Stenotrophomonas maltophilia*	0	0.00%	0	0.00%	0	0.00%	1	2.27%
	*Klebsiella* spp.	1	2.27%	0	0.00%	2	4.55%	1	2.27%
	*Pseudomonas* spp.	0	0.00%	1	2.27%	2	4.55%	2	4.55%
	*Serratia marcescens*	0	0.00%	0	0.00%	0	0.00%	1	2.27%
	*Morganella morganii*	0	0.00%	0	0.00%	0	0.00%	1	2.27%
	*Staphylococcus aureus*	0	0.00%	1	2.27%	4	9.09%	5	11.36%
	*Streptococcus* spp.	0	0.00%	0	0.00%	1	2.27%	3	6.82%
*Fungal infections*	*Candida albicans*	1	2.27%	0	0.00%	5	11.36%	4	9.09%
	*Candida* nonalbicans	1	2.27%	1	2.27%	6	13.64%	13	29.55%

**Table 6:** Mold-associated infections in COVID-19 patients.

Microorganism		SARS-CoV-2 Positive (*n* = 14)	
		*n*=	Percentage
*Bacterial* *infections*	*Citrobacter* spp.	1	7.14%
	*Enterobacter* spp.	1	7.14%
	*Enterococcus* spp.	1	7.14%
	*Klebsiella* spp.	1	7.14%
	*Pseudomonas* spp.	2	14.29%
	*Staphylococcus aureus*	1	7.14%
	*Streptococcus* spp.	1	7.14%
*Fungal* *infections*	*Candida albicans*	4	28.57%
	*Candida* non-albicans	4	28.57%

**Table 7:** Risk factors (comorbidities) that were present in patients with mold infections based on age group.

Comorbidities	Child		Youth		Adult		Senior		Grand Total
	*n*=	%	*n*=	%	*n*=	%	*n*=	%	
Cardiovascular system	0	0.00%	0	0.00%	3	4.41%	14	20.59%	17
Central nervous system	0	0.00%	0	0.00%	1	1.47%	2	2.94%	3
COVID-19	0	0.00%	0	0.00%	3	4.41%	11	16.18%	14
Diabetes mellitus	0	0.00%	0	0.00%	1	1.47%	2	2.94%	3
Digestive system	1	1.47%	1	1.47%	1	1.47%	5	7.35%	8
Ear-nose-throat	0	0.00%	0	0.00%	0	0.00%	1	1.47%	1
Kidneys	0	0.00%	0	0.00%	3	4.41%	3	4.41%	6
Malignities	0	0.00%	0	0.00%	3	4.41%	2	2.94%	5
Respiratory system	1	1.47%	1	1.47%	11	16.18%	12	17.65%	25
Skin	0	0.00%	0	0.00%	0	0.00%	7	10.29%	7
Total	2	2.94%	2	2.94%	26	38.24%	59	86.76%	-

**Table 8:** Comorbidities in COVID-19 patients who developed filamentous mycoses.

Comorbidities	SARS-CoV-2 Positive (*n* = 14)	
	*n*=	% from Positive Samples
Cardiovascular system	9	64.29%
Respiratory system	7	50.00%
Diabetes	3	21.43%
Kidneys	3	21.43%
Central nervous system	1	7.14%
Ear-nose-throat	1	7.14%
Skin	1	7.14%
Digestive system	2	14.29%

## Data Availability

Data are available upon request from authors.
